# Learning the optimal scale for GWAS through hierarchical SNP aggregation

**DOI:** 10.1186/s12859-018-2475-9

**Published:** 2018-11-29

**Authors:** Florent Guinot, Marie Szafranski, Christophe Ambroise, Franck Samson

**Affiliations:** 1 UMR 8071 LaMME – UEVE, CNRS, ENSIIE, USC INRA, 23 bd de France, Evry, 91000 France; 2 BIOptimize, Reims, 51000 France; 3 UMR MIA-Paris – AgroParisTech, INRA, Université Paris-Saclay, Paris, 75005 France

**Keywords:** Genome-wide association study, Statistical genetics, Variable selection, Hierarchical clustering

## Abstract

**Background:**

Genome-Wide Association Studies (GWAS) seek to identify causal genomic variants associated with rare human diseases. The classical statistical approach for detecting these variants is based on univariate hypothesis testing, with healthy individuals being tested against affected individuals at each locus. Given that an individual’s genotype is characterized by up to one million SNPs, this approach lacks precision, since it may yield a large number of false positives that can lead to erroneous conclusions about genetic associations with the disease. One way to improve the detection of true genetic associations is to reduce the number of hypotheses to be tested by grouping SNPs.

**Results:**

We propose a dimension-reduction approach which can be applied in the context of GWAS by making use of the haplotype structure of the human genome. We compare our method with standard univariate and group-based approaches on both synthetic and real GWAS data.

**Conclusion:**

We show that reducing the dimension of the predictor matrix by aggregating SNPs gives a greater precision in the detection of associations between the phenotype and genomic regions.

## Background

### Context

Recent breakthroughs in microarray technology have meant that hundreds of thousands of single nucleotide polymorphisms (SNPs) can now be densely genotyped at moderate cost. As a result it has become possible to characterize the genome of an individual with up to a million genetic markers. These rapid advances in DNA sequencing technologies have also made it possible to carry out exome and whole-genome sequencing studies of complex diseases. In this context, Genome-Wide Association Studies (GWAS) have been widely used to identify causal genomic variants implied in the expression of different human diseases (rare, Mendelian, or multifactorial diseases).

From a statistical point of view, looking for these variants can be supported by hypothesis testing. The standard approach in GWAS is based on univariate regression (logistic regression in case-control studies), with affected individuals being tested against healthy individuals at one or more loci. Classical testing schemes are subject to false positives (that is to say SNPs that are falsely identified as significant variables). One way around this problem is to apply a correction for the False Discovery Rate [1, 2]. Unfortunately, this increases the risk of missing true associations that have only a small effect on the phenotype, which is usually the case in GWAS. [3] suggested that standard approaches such as multiple hypothesis testing may not be appropriate for the detection of small effects from multiple SNPs. In such cases a significant part of the heritability can be missing and GWAS fails to detect all possible genetic variants associated with a disease.

Furthermore, this kind of standard approach faces other limitations:

1. It does not directly account for correlations among the predictors, whereas these correlations can be very strong as a result of linkage disequilibrium (LD). SNPs can be correlated even where they are not physically linked, because of population structure or epistasis (gene by gene interactions).
2. It does not account for epistasis, i.e. causal effects that are only observed when certain combinations of mutations are present in the genome.
3. It does not directly provide predictive models for estimating the genetic risk of the disease.
4. It focuses on identifying common markers with allele-frequency (MAF) above 5%, although it is likely that analyzing low-frequency (0.5*%*< MAF <5*%*) and rare (MAF <0.5*%*) variants would be able to explain additional disease risks or trait variability [4].

Uncovering some of the missing heritability can sometimes be achieved by taking into account correlations among variables, interaction with the environment, and epistasis, but this is rarely feasible in the context of GWAS because of the multiple testing burden and the high computational cost of such analyses [5]. In the context of rare-variant association analysis, a number of region- or gene-based multimarker tests have been proposed as burden tests [6], variance-component tests [7] or combined burden and variance component tests [8]. Instead of testing each variant individually, these methods evaluate the cumulative effects of multiple genetic variants in a gene or a region, increasing power when multiple variants in the group are associated with a given disease or trait.

Furthermore, regarding limitation (4), analyzing rare variants is more complex than analyzing more common variants and a large sample size is needed to observe a rare variant with a high probability.

### Group and aggregation based methods for common variants

Although classical GWAS have limitations that prevent a full understanding of the heritability of genetic and/or multifactorial diseases, there are nevertheless ways of overcoming these limitations to some degree. For instance, it is possible to take into account the structure of the data in the hypothesis testing procedure. As an illustration, [9] proposed a hierarchical testing approach which considers the influence of clusters of highly correlated variables rather than individual variables. The statistical power of this method to detect relevant variables at single SNPs level was comparable to that of the Bonferroni-Holm procedure [10], but the detection rate was much higher for small clusters, and it increased further at coarser levels of resolution.

Group-based methods require an appropriate group definition. In GWAS, the usual approach is to group SNPs which are included in the same gene but this limits the analysis to coding regions. It is well known that the human genome is structured into haplotype blocks, i.e. sizable regions over which there is little evidence for historical recombination and within which only a few common haplotypes may be observed [11]. The boundaries of blocks and the specific haplotypes that they contain are highly correlated across populations [12]. With this property of the human genome in mind, [13] developed a method for detecting haplotype-disease associations in genome-wide studies, based on sliding windows of adjacent SNPs, along with a Monte Carlo procedure to adjust for multiple testing.

In [14], the authors proposed to group SNPs into sets on the basis of their proximity to genomic features such as genes or haplotype blocks and then to identify the joint effect of each set via a logistic kernel-machine-based test. This approach lays the foundation for the Sequence Kernel Association Test method [7].

In the broad family of linear models, [15] introduced a likelihood ratio-based set test that accounts for confounding structure. The model is based on the linear mixed model and uses two random effects, one to capture the set association signal and one to capture confounders. They demonstrate a control of type I error as well as an improved power over more traditionally used score test. Other methods focus on multiple linear regression either by taking into account the linkage disequilibrium within the genes to improve power [16] or by clustering variants with weak association around known loci to increase the percentage of variance explained in complex traits [17].

Finally, other approaches will focus on the aggregation of summary statistics of single SNPs within a same gene with for instance the data-driven aggregation of summary statistics described in [18] or the procedures of *p*-value combination in [19]. In the cited articles, the methods are used on SNPs located in coding region (or extended intronic region in [19]) but can be extended to any set of SNPs as long as we pre-specified a set of variants within a region. However the power for each test remains dependent of the true disease model. Furthermore, this kind of approaches may also lose statistical power in comparison to single-variant-based tests when only a very small number of the variants in a gene are associated with the trait, or when many variants have no effect or causal variants are low-frequency variants [4].

### Organisation of the paper

The present paper proposes a block-wise approach for GWAS analysis which leverages the LD structure among the genomic variants to reduce the number of hypotheses testing. We aggregate the SNPs into different clusters according to their LD levels and use a supervised learning approach to identify the clusters of SNPs related to a case-control phenotype. Our algorithm provides a group structure for the variables, enabling us to define a function that aggregates these clusters into new variables to be used in the GWAS machinery. The advantage of this method is that aggregating clusters of several SNPs into a single variable reduces the dimension of the data without loss of information, since we are grouping variables that are highly correlated (in strong LD).

We compare our method in different scenarios with the baseline approach, i.e. univariate hypothesis testing [20] and with a state-of-the-art method, the logistic kernel machine method developed by [14] on both synthetic and real datasets from the Wellcome Trust Case Control Consortium [21] and on ankylosing spondylitis data [22].

## Method

In this section we describe a new method for performing GWAS using a four-step method that combines unsupervised and supervised learning techniques. This method improves the detection power of genomic regions implied in a disease while maintaining a good interpretability. This method consists in:

1. Performing a spatially constrained Hierarchical Agglomerative Clustering (constrained-HAC) of the SNPs matrix X using the algorithm developed by [23].
2. Applying a function to reduce the dimension of X using the group definition from the constrained-HAC. This step is described and illustrated in Fig. 1.

**Fig. 1:**
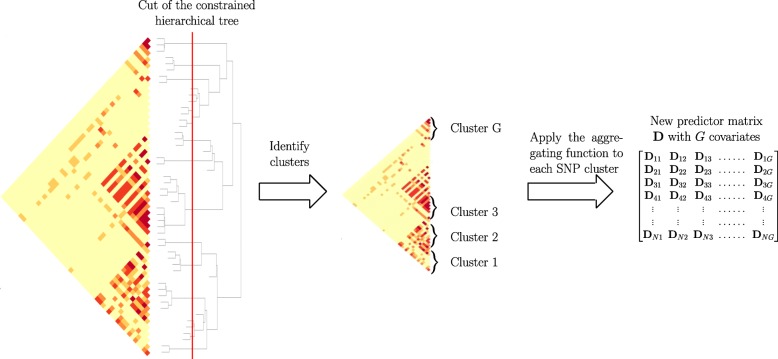
Schematic view of Step 2 of the algorithm to calculate the matrix of predictors D
3. Estimating the optimal number of groups using a supervised learning approach to find the best cut into the hierarchical tree (cut level algorithm). This algorithm combines Steps 1 and 2 into an iterative process.
4. Applying the function defined in Step 2 to each group identified in Step 3 to construct a new covariate matrix and perform multiple hypothesis testing on each new covariate to find significant associations with a disease phenotype *y*.

### Step 1. Constrained-HAC

In GWAS, the covariates are ordinal and correspond to SNP genotypes such that X_*ij*_∈{0,1,2} corresponds to the number of minor alleles at locus *j*∈[1,…,*J*] for observation *i*∈[1,…,*N*].

To take into account the structure of the genome in haplotype blocks, we group the predictors (SNPs) according to their LD in order to create a new predictor matrix which reflects the structure of the genome. We first use the algorithm developed by [23], which clusters SNPs into adjacent blocks. The clustering method is a spatially constrained hierarchical clustering based on Ward’s incremental sum-of-squares algorithm [24], in which the measure of dissimilarity is not based on the Euclidean distance but rather on the linkage disequilibrium between two SNPs: 1−*r*^2^(*j*,*j*^′^). The algorithm also makes use of the fact that the LD matrix can be modeled as block-diagonal by allowing only groups of variables that are adjacent on the genome to be merged, which significantly reduces the computation cost. This algorithm is available via the R package called adjclust on https://cran.r-project.org/web/packages/adjclust.

### Step 2. Dimension reduction function

One way of addressing issues related to high-dimensional statistics (and in particular the multiple testing burden that we mentioned above) is to reduce the dimensionality of the predictor matrix $ \mathrm {X} \in \mathbb {R}^{N \times J} $ by creating a reduced matrix D with new covariates that nevertheless remain representative of the initial matrix. This means reducing the number of predictors *J* to *G*≪*J*, with row D_*i*._ the *G*-dimensional vector of new predictors for observation *i*. In this study we use a blockwise approach to construct a matrix of new uncorrelated predictors $ \mathrm {D} \in \mathbb {R}^{N \times G} $, with *G* the number of groups in linkage disequilibrium identified via the constrained agglomerative hierarchical clustering described in Step 1.

While classical methods use the initial set of covariates to predict a phenotype, we propose combining a clustering model with a dimension reduction approach in order to predict *y*. For each group identified with the constrained-HAC, we apply a function to obtain a single variable defined as the number of minor alleles present in the group. For each observation *i* and in each cluster *g*∈[1,…,*G*], the variable is defined as:

1 $$  \mathrm{D}_{ig}~=~\sum\limits_{j \in g} \mathrm{X}_{ij}.  $$

In order that the values for the different groups are comparable, we eliminate the effect of group size by centering and scaling the matrix D to unit variance. In the remainder of the paper we will refer to the covariates in D as *aggregated-SNP* variables.

### Step 3. Optimal number of groups estimation

Estimating the optimal number of groups to select, i.e. the level at which the hierarchical clustering tree should be cut, is a fundamental matter which impacts the relevance of the association analysis. It is known that the human genome is structured into haplotype blocks with little or no within-block recombination [12], but it is not easy to determine how these blocks are allocated throughout the genome for a given set of SNPs.

In the literature, in an unsupervised learning context, a number of models have been proposed for determining this optimal number of groups [25–28]. These methods are all based on the measure of within-group dispersion *W*_*G*_ with *G*∈[1,…,*P*]. Since GWAS consist in evaluating the likelihood of the disease from genetic markers, we propose using the phenotype *y* as a way of determining the optimal number of clusters.

We propose here a supervised validation set approach to find this optimum. Since this algorithm aims to identify phenotype-related SNPs clusters, it is necessary to split the dataset into two subsets to avoid an inflation of type I errors in the testing procedure. One subset, [*Y*_*sub*1_,X_*sub*1_], is used to choose the optimal cut and the second one, [*Y*_*sub*2_,X_*sub*2_], to perform the hypothesis testing in Step 4.


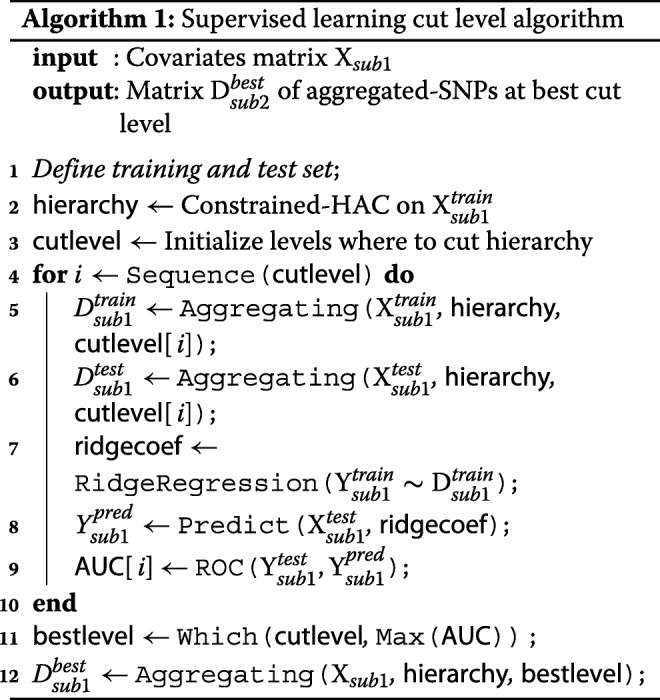


First we apply the constrained-HAC on a subset $\mathrm {X}^{train}_{sub1} \subset \mathrm {X}_{sub1}$, and for a given level of the hierarchy we apply the dimension reduction function defined above (Step 2) to each of the *G* clusters to construct the matrix $\mathrm {D}^{train}_{sub1}$. We then fit a ridge regression model to estimate the coefficients of the predictors in $\mathrm {D}^{train}_{sub1}$. Ridge regression is a penalized model which shrinks the estimated coefficients towards zero and is known to have a good stability in comparison to other penalized-regression models such as lasso regression [29]. Moreover, a link can be established between the ridge regression model and the mixed linear model used in the estimation of the heritability in a high-dimensional setting [30]. Once the coefficients are estimated, we predict the phenotypic values on the test set using the matrix $\mathrm {D}^{test}_{sub1}$ and calculate either the mean test set error when the phenotype is quantitative or the Area Under the ROC curve (AUC-ROC) when it is binary. The procedure, summarized in Algorithm 1, is then repeated for different levels in the hierarchy and the optimal cut level in the tree is defined as the level which maximizes the prediction accuracy criterion.

### Step 4. Multiple testing on aggregated-SNP variables

Once the optimal number of groups has been determined, we apply the function (1) to each selected group and construct the matrix of *aggregated-SNP*. Here we use a standard Single Marker Analysis (SMA) to find associations with the phenotype, but instead of calculating *p*-value for each SNPs in X_*sub*2_, we calculate *p*-value for each *aggregated-SNP* variables in D_*sub*2_.

As in standard SMA, a univariate generalized linear model [31] is fitted for each variable D_.*j*_: *f(μ*_*i*_*)*=D_*ij*_*β*, where $\mu _{i} \equiv \mathbb {E}(\mathrm {Y}_{i}|\mathrm {D}_{i}) $ ($\mathrm {Y_{i}} \thicksim \text {some exponential family distribution})$, *f* is a smooth monotonic ’link function’, D_*ij*_ is the *i*^*th*^ row of the model matrix D_.*j*_ of aggregate-SNP and *β* is a vector of 2 unknown coefficients with *β*_0_ for the intercept and *β*_1_ for the predictor *j*. Where the response variable is a binary trait (i.e. case-control phenotype), we use the logit function as the ’link function’ *f* and $ Y_{i} \thicksim \text {Bernoulli distribution}$. This model is known as the logistic regression model. Then, for each single-predictor model, we perform a Likelihood Ratio Test where we compare the intercept-only model against the single-predictor model and get for each predictor a *p*-value using the $\tilde \chi ^{2}$ distribution.

Given that a large number of covariates are being tested, we need to compute an appropriate significance threshold to control the family-wise error rate, $FWER = \mathbb {P}(FP > 1)$, with *FP* being the False Positive, since keeping the threshold at the conventional value of *α*=0.05 would yield numerous false positives. Several approaches, including the Bonferroni correction, have been proposed in the context of genetic studies for controlling the FWER [32]. An alternative approach, developed by [1], seeks to control the False Discovery Rate (FDR) which is the expectation of ratio between the number of false positives and the total positive outcomes: ${FDR = \mathbb {E}\left (\frac {FP}{FP + TP}\right)}$, with *TP* being the True Positive. The Bonferroni correction reduces the significance level according to the number of tests carried out in the study. However, in the context of GWAS, where hundreds of thousands of tests have to be performed, the Bonferroni correction is too strong, and will often decrease the significance threshold to a level where almost nothing is significant. Controlling FDR is therefore preferable. It is an approach that is less stringent but nonetheless powerful. The method for controlling FDR does not directly set a significance threshold, but rather identifies the largest *p*-value that is substantially smaller than its expected value (by a factor of at least 1/ *ϕ* where *ϕ* is the desired FDR level), given that all the tests follow *H*_0_. The *p*-value thus identified and all smaller *p*-values are deemed to be significant.

## Numerical simulations

The performance evaluation described below was designed to assess the ability of our method to retrieve causal SNPs or causal clusters of SNPs under different simulation scenarios. For each scenario, we use a matrix *X*_*HAPGEN*_ of SNPs generated by the HAPGEN2 software [33] with a sample size of 1000 individuals. This software allows to simulate an entire chromosome conditionally on a reference set of population haplotypes (from HapMap3) and an estimate of the fine-scale recombination rate across the region, so that the simulated data share similar patterns with the reference data. We generate the chromosome 1 (103 457 SNPs) using the haplotype structure of CEU population (Utah residents with Northern and Western European ancestry from the CEPH collection) as reference set. The HAPGEN2 software allows to generate a controls-only matrix of SNPs (no disease allele). We filtered this matrix according to the minor allele frequency to only keep SNPs with a MAF greater than 5% thus reducing the size of X_HAPGEN_ to 60 179 SNPs.

We generate a posteriori the phenotype using the logit model with a given set of causal SNPs or cluster of SNPs. The main difference between the different scenarios is to be found in the way that the case-control phenotype y is simulated.

### Simulation of the case-control phenotype

For all simulation scenarios, we simulated a case-control phenotype *y* under a logistic regression model. The case-control phenotype is generated following a Bernoulli distribution function, following the conditional probability $\mathbb {P}\left (y_{i}=1|\tilde {\mathrm {X}}_{i.}\right)$ with $\tilde {\mathrm {X}} \in \mathbb {R}^{n \times \ell }$ a matrix constructed by sampling *ℓ* causal variables from X_HAPGEN_, $\tilde {\mathrm {X}}_{i.}$ being the *ℓ*-dimensional vector corresponding to the *i*^*th*^ observation.

The conditional probability is calculated using the logit model:

$$\mathbb{P}\left(y_{i}=1|\tilde{\mathrm{X}}_{i.}\right) = \frac{\exp\left(\beta_{0} + \boldsymbol\beta \tilde{\mathrm{X}}_{i.}\right)} {1 + \exp\left(\beta_{0} + \boldsymbol\beta \tilde{\mathrm{X}}_{i.}\right)}, $$

where ***β***=[*β*_1_,…,*β*_*ℓ*_] is the vector of coefficients corresponding to the *ℓ* predictors $\left [\tilde {\mathrm {X}}_{.1}, \dots, \tilde {\mathrm {X}}_{.\ell }\right ]$ and *β*_0_ is the intercept defined as ${ ln\left (\frac {\pi }{(1-\pi)}\right)}$, with *π* the true prevalence of the disease in the population. The predictors are centered to have zero-mean before generating the vector of probability.

One way to have an association between the response and the predictors strong enough to be detected is to set large ***β*** coefficients on the predictors. Indeed there is a direct relationship between the odd ratio (*OR*) of a covariate $\tilde {\mathrm {X}}_{i.}$ and its corresponding coefficients ***β***_*i*_ in the logistic regression model [34] given by $\phantom {\dot {i}\!}OR_{i} = e^{(\boldsymbol \beta _{i})}$. In our simulations, the difficulty of the problem, i.e. the power to detect an association, is linked to the number of causal predictors used to generate *y* and the *OR* set to each predictors.

To simulate different scenarios we considered the following parameters:

1. Nature of the causal predictors:
  **Clusters of SNP:** For each replicates, *ℓ*=1,2,3 genomic regions have been identified to be causal. These regions have been chosen among the matrix *X*_*HAPGEN*_ to have different levels of LD among the SNPs that compose them. The average correlation coefficient among the SNPs in these regions varies from *r*^2^=0.6 to *r*^2^=0.85 and the size of the region varies from 20 SNPs to 60 SNPs. Once identified, the causal regions were aggregated using the function described in Step 2 to construct a matrix $\tilde {\mathrm {X}}$ of *aggregated-SNPs* predictors used to generate the case-control phenotype. Under this scenario, all the SNPs within the region in LD can be considered as causal but it is the aggregated variables that is used to generate the phenotype. We will refer to it as the *SNPclus* scenario.
  **Single SNPs.** In this scenario the phenotype was simulated by directly using sampled SNPs from the same causal regions identified in the *SNPclus* scenario. For each replicates, we chose 10 individuals SNPs among each of these regions to construct a matrix $\tilde {\mathrm {X}}$ with *ℓ*=10,20,30 single SNPs predictors, depending on the number of causal regions. This matrix is then used to generate the case-control phenotype. The chosen SNPs have a MAF varying from 10*%* to 30%. We will refer to this scenario as the *singleSNP* scenario.
2. Number of causal predictors *ℓ* and number of replicates:
  We performed 5 replicates for each combination *ℓ*× number of scenarios and we evaluate the average performance over these 5 replicates. For each scenario we considered from 1 to 3 causal genomic regions, thus, for *SNPclus* scenario, we used up to 3 causal predictors, and for the *singleSNP* scenario, up to 10×3=30 causal predictors to generate the case-control phenotype.
3. Odd ratio (*β* coefficients) of the causal predictors:
  For the *SNPclus* scenario we chose an equal OR of 2.7 for each causal aggregated predictors, corresponding to a ***β*** coefficient equal to 1. For the *singleSNP* scenario we chose an equal OR of 1.1 for each causal predictors, corresponding to a ***β*** coefficient equal to 0.1. The rationale behind these coefficients arises from the hypothesis that the combined effect of several low-effect SNPs on the phenotype is stronger than the effects of each individual SNP.

As previously mentionned, we generated the phenotype using causal SNPs simulated with the HAPGEN2 software. However, as commercial genechips such as Affymetrix and Illumina arrays do not genotype the full sequence of the genome, some SNPs are thereby unmapped and the marker density is in general lower than the HapMap marker density. That is why we chose, in our numerical simulation, to generate the phenotype with causal variables chosen from the HAPGEN matrix and to assess the performance of the methods using only those SNPs which are mapped on a standard Affymetrix genechip (about 23 823 mapped SNPs). By doing so, some causal SNPs are not mapped on the commercial SNP set and thus simulations are more similar to real genome-wide analysis conditions.

## Results

### Performance evaluation

**Competitors** The objective of our method being to identify the optimal scale at which to perform association studies, we compared our proposal with several methods working at different genomic scales. The purpose is to assess the ability of each method to retrieve true causal genomic regions in the different simulation scenarios. For each scenario, four approaches have been considered:

- SKAT*tree*, a SKAT model using our group definition,
- SKAT*notree*, a SKAT model using an alternative group definition produced by successive chunks of 20 SNPs,
- SMA, the classical Single Marker Analysis,
- SASA (Single *Aggregated-SNP* Analysis) a method close to SMA, where instead of testing the genotype-phenotype association using each single SNP, we are testing it using *aggregated-SNP* variables.

The two above described group definitions for SKAT were considered to evaluate the impact of the group structure on the association findings.

The comparison with SMA allows to highlight the advantage of working at a group scale. We hypothesize that grouping low-effect SNPs should have a better statistical power than testing the main effects at single-SNP level.

For all methods, we compare the results using 2 types of multiple testing corrections : the methods of Holm-Bonferroni [10] and [1].

**True and False Positive definitions.** The problem of retrieving true causal associations can be represented as a binary decision problem where the compared methods are considered as classifiers. The decision made by a binary classifier can be summarized using four numbers: True Positives (*TP*), False Positive (*FP*), True Negatives (*TN*) and False Negatives (*FN*). We represent True Positive Rate (Recall or Power=*TP*/(*FN*+*TP*)) versus Precision (Precision=*TP*/(*FP*+*TP*)).

In this context, a True Positive corresponds to a true causal genomic region associated to significant *p*-value. The definition of what can be considered as the true causal genomic region may nevertheless be subject to some ambiguity. In GWAS, the presence of LD between SNPs often leads to consider the signal associated to multiple neighboring SNPs as indicating the existence of a single genomic locus with possible influence on the phenotype.

In our simulations, a causal genomic region is defined a priori as a causal predictor in the logit model. However, since the clusters of SNPs identified by our algorithm are not totally independent, some residual correlation may remain between clusters. This leads to question the notion of relevant variable when the variables are structured into strongly correlated groups. Should all the variables of the block be considered as explanatory, or should we define as only true positives the causal variables used to generate the phenotype ?

In order to circumvent this issue, we chose to relax the definition of a False Positive joining the work of [35] and [36] where they propose to control the FDR in GWAS by considering significiant SNPs correlated to the true causal variables as true positives.

For the simulation of the phenotype, we hypothesize an underlying multivariate regression model, but test for univariate model as it is the usual practice, which leads to reconsider the definition of true positive. As in [36] we consider the set of true positive as the union of the causal true positive and the linked true positive, which are regions adjacent to the causal regions and correlated with them at a level of at least 0.5. Regarding the single-marker analysis approach, since it works at the single SNP level, we compare it with the others in the *singleSNP* scenario only.

### Results and discussions of the numerical simulations

**Area Under the ROC Curve.**

**Fig. 2:**
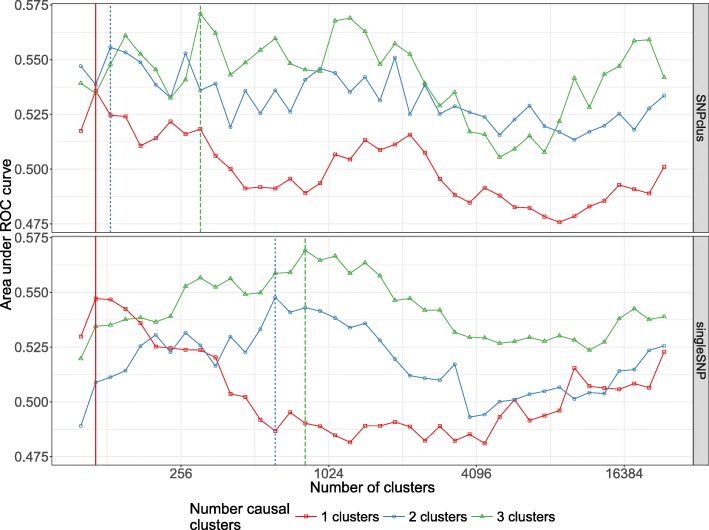
Area under ROC curves according to the number of clusters in the *clusSNP* and *singleSNP* scenarios: the vertical lines indicate the number of aggregated-SNPs (clusters) obtained with Algorithm 1, i.e. the level where the prediction error is minimized (AUC-ROC at its maximum)

For each simulation, the cut level algorithm was applied. We recall that this algorithm calculates a prediction error on a test set for several levels in a constrained-HAC tree with a ridge regression model and chooses the level for which this error is the smallest. The AUC-ROC is plotted for the different levels, and the best cut level corresponds to the level for which AUC-ROC is the greatest. The results from the simulation scenario *clusSNP* and *singleSNP* described in “Simulation of the case-control phenotype” section are shown in Fig. 2. Our algorithm cuts the hierarchy either at a fairly high level (few large clusters) or at a low level (many small clusters), depending on the number of causal variables we used to generate the phenotype. The more the number of causal regions decreases, the higher the algorithm cuts in the hierarchical tree. In either case our algorithm is able to increase the predictive power by aggregating SNPs with the function (1).

We are thus able build a matrix of uncorrelated aggregated-SNP predictors that are representative of the initial SNP matrix and strongly linked to the phenotype.

**Performance results for simulated data.** As described in the performance evaluation section, we evaluate and compare the methods using two metrics, namely Recall or Power=*TP*/(*FN*+*TP*) and Precision=*TP*/(*FP*+*TP*).

Here the *Precision* metric is somewhat relaxed compared to its true definition since we adapted the definition of a True Positive and False Positive to the GWAS context. It is important to note that for all the methods, we compare the Benjamini-Hochberg method to control FDR with the Bonferroni correction to control FWER at a threshold of 5%. However, since there are residual correlations between SNPs clusters and that the replication of numerous samples per combination of parameters is difficult in this realistic setting of simulations, the observed Type I error rate may be greater than 5%. What we think is important to put forward to in these simulations is the ability of our algorithm to define groups of relevant clusters that will be detected on average with more precision and more power (SASA and SKAT*tree*) than using an arbitrary group definition (SKAT*notree*) or no definition of groups at all (SMA).

**Fig. 3:**
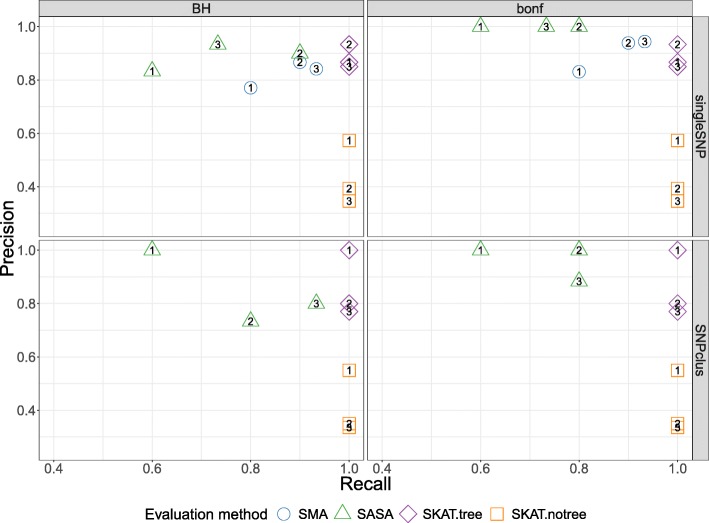
Recall vs Precision for each method (shape and colors in plot). In rows are the simulation scenarios. In columns, we evaluate performance using Benjamini-Hochberg threshold (left) and bonferroni correction threshold (right). The second row illustrates the performance to retrieve the true causal genomic region under the *SNPclus* scenario, thus only group-based approaches are considered (SASA, SKAT.tree and SKAT.notree). The numbers inside the points correspond to the number of causal predictors and each point is the average value of 5 replicates

The results represented in Fig. 3 show that the methods using our algorithm for the cluster definition (SASA and SKAT*tree*) have in average a better precision than the two other methods. The approach SASA, which combine our clustering algorithm and the aggregating function (1) to test the association of aggregated-SNPs with the phenotype, perform poorly in term of Recall but is far better in term of Precision compared to SMA and SKAT*notree*. These results suggest that it is better to combine our algorithm with the SKAT method than with the SASA method. We also note that applying the SKAT approach on an arbitrary group definition (SKAT*notree*) lead to a good recall but a very poor precision, showing the benefit of using our custom group definition in this context. Regarding the SMA approach in the *singleSNP* scenario, we can observe a loss in term of Recall compare to the SKAT*tree* and SKAT*notree* method suggesting that we can take benefit of grouping low effect SNPs to improve the power to detect causal genomic regions.

In GWAS, having a method with a good precision is as important, or even more important, than having a good recall. It is better to spot a few significant associations with a high certainty than to spot numerous significant associations but with only a low level of certainty for most of them. For this reason, we believe that our method represents an improvement in terms of precision without loss of power insofar as SKAT*tree* seems able to detect significant genomic regions associated with the phenotype with a higher degree of certainty than standard approaches.

### Application in wellcome trust case control consortium(WTCCC) and Ankylosing Spondylitis (AS) studies

To evaluate the performance of our method on real data, we performed GWAS analysis on datasets made available by [21]. The WTCCC data collection contains 17000 genotypes, composed of 3000 shared controls and 14000 cases representing 7 common diseases of major public health concern: inflammatory bowel disease (IBD), bipolar disorder (BD), coronary artery disease (CAD), hypertension (HT), rheumatoid arthritis (RA), and Type I (T1D) and Type II (T2D) diabetes. Individuals were genotyped with the Affymetrix GeneChip 500K Mapping Array Set and are represented by about 500,000 SNPs (before the application of quality control filters).

In parallel to the analysis of the WTTCC data, we decided to assess our method on another dataset from a different study. The ankylosing spondylitis (AS) dataset consists of the French subset of the large study of the International Genetics of Ankylosing Spondylitis (IGAS) study [22]. For this subset, unrelated cases were recruited through the Rheumatology clinic of Ambroise Paré Hospital (Boulogne-Billancourt, France) or through the national self-help patients’ association: “Association Française des Spondylarthritiques”. Population-matched unrelated controls were obtained from the “Centre d’Etude du Polymorphisme Humain”, or were recruited as healthy spouses of cases. The dataset contains 408 cases and 358 controls, and each individual was genotyped for 116,513 SNPs with Immunochip technology.

To remove the bias induced by population stratification in Genome-Wide analysis, we added the first 5 genomic principal components into the regression model as described in [37]. Since the methods evaluated here do not deal with missing values, we chose to impute the missing genotypes with the most frequent genotypic value, *h*_*j*_ observed for each *j* SNP.

**Fig. 4:**
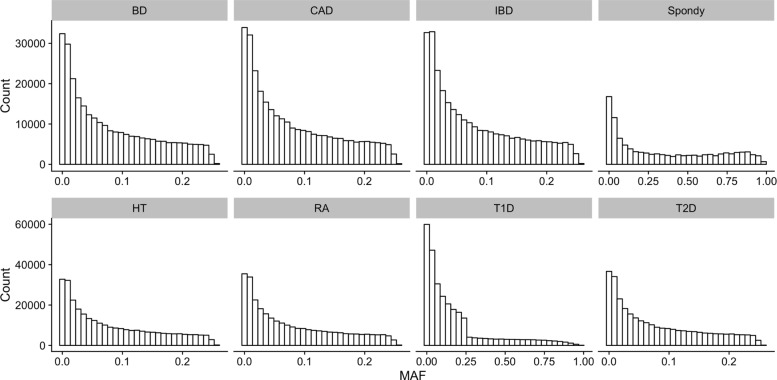
Histograms of Minor Allele Frequencies (MAF) distribution in each datasets. (BD) Bipolar disorders; (CAD) Coronary arthery disease ; (IBD) Inflammatory bowel disease ; (HT) Hypertension ; (RA) Rheumatoid arthritis ; (T1D) Type I diabetes ; (T2D) Type II diabetes

For each each dataset, we filtered the values to keep only those SNPs having a MAF greater than 5%. The minor allele frequencies of each datasets are represented in Fig. 4.

We applied our cut level algorithm to find relevant clusters of SNPs and we performed single marker analysis on single SNPs (SMA) and on groups of SNPs (SASA, SKAT*tree*, SKAT*notree*). We then compared the significant associations detected by the different methods to reveal possible new associations with the phenotype.

### Results in WTCCC and AS studies

**AUC-ROC curves**

In this section, we compare the AUC-ROC curves generated by our cut level algorithm for each disease (WTCCC and AS data).

**Fig. 5:**
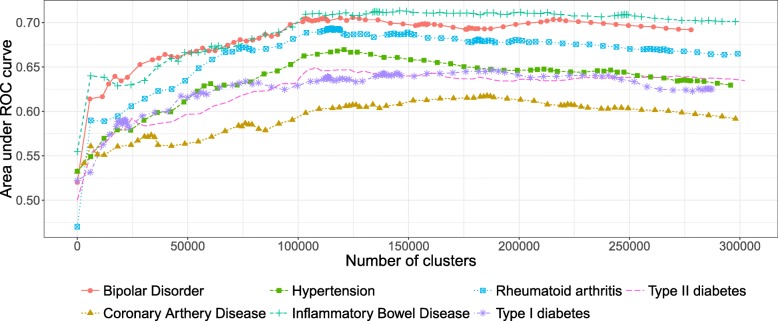
AUC-ROC for different cut levels in a HAC-tree of 7 WTCCC diseases after quality control filters. Each point corresponds to an AUC value computed on a test set from a logistic ridge regression model for a given level in the constrained-HAC tree

Concerning the WTCCC diseases, given that patients were all genotyped using the same GeneChip, their genotypes have the same LD structure, and therefore the shapes of the AUC-ROC curves should be very similar between the different diseases. As can be observed in Fig. 5 (WTCCC diseases), the shape of the AUC-ROC curves are closely similar, with a chosen cut level located around 100 000 clusters of SNPs, suggesting a shared LD pattern among patients.

**Fig. 6:**
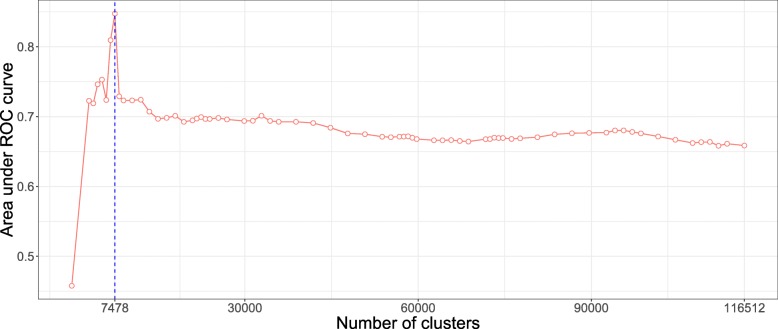
AUC-ROC for different cut levels in a HAC-tree of the spondylitis arthritis disease (Immunochip genechip). Each point corresponds to an AUC value computed on a test set from a logistic ridge regression model for a given level in the constrained-HAC tree

In contrast, the AUC-ROC from the AS data (Fig. 6) behaves differently from the WTCCC data. Predictive power is substantially improved if *aggregated-SNP* predictors are used at a fairly high level in the hierarchical tree (7478 optimal clusters identified by the cut level algorithm). It is relevant to note that the pattern we observe on this real dataset is similar to the pattern we observed in the numerical simulations, especially under the *clusSNP* scenario.

**Fig. 7:**
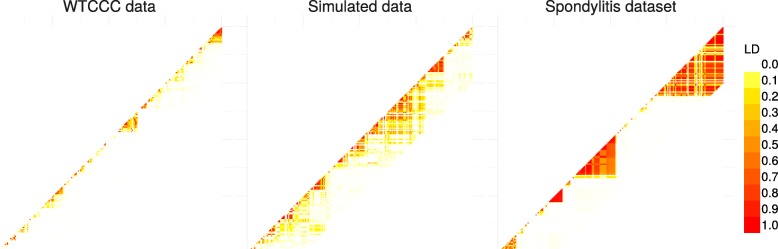
Comparison of linkage disequilibrium level among SNPs for 3 different types of dataset: WTCCC, simulated and ankylosing spondylitis datasets. LD computation is based on *R*^2^ between SNPs

As we remarked concerning the WTCCC results, the algorithm identifies a relatively high number of clusters in relation to AS and simulated data. This difference is certainly due to the LD level among the genetic markers in the Affymetrix GeneChip. The correlation levels among SNPs for a given bandwith are similar between the simulated and the AS data, but greater than for the WTCCC data (Table 1 and Fig. 7). This suggests that there is a stronger LD pattern between blocks of SNPs in AS and simulated data, implying that the optimal number of clusters identified by the algorithm is dependent on the LD level among variables.

**Table 1:** Comparison of marker density and averaged LD level between markers in a region of 300 SNPs for the different datasets

*Dataset*	*SNP/kb*	Median	Mean
Simulated data	1.3×10^−27^	1×10^−2^	0.11
WTCCC data	7×10^−32^	9×10^−4^	0.03
AS data	9×10^−9^	3×10^−2^	0.27

**GWAS analysis on AS and WTCCC datasets**

**Fig. 8:**
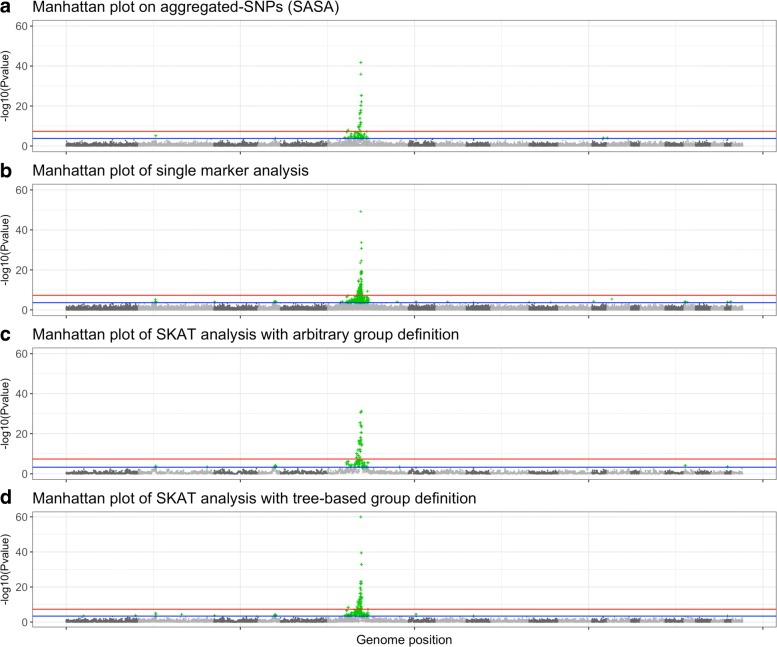
Manhattan plots showing results of GWAS analysis on ankylosing spondylitis data. For each Manhattan plot, the Benjamini-Hochberg (BH) threshold is represented by the blue line and the Bonferroni threshold by the red line. According to the BH threshold, there are: (**a**) 64 significantly associated aggregated-SNPs; (**b**) 602 significantly associated single SNP; (**c**) 80 significantly associated groups of SNPs and (**d**) 138 significantly associated groups of SNPs

To evaluate the ability of our procedure to discover new associations between SNPs and ankylosing spondylitis, we compare our procedure with the univariate approach (SMA) and SKAT model with our group definition and arbitrary group definition (20 SNPs). For SASA, we perform multiple hypothesis testing on the aggregated-SNP predictors in order to unravel significant associations with the phenotype. Fig. 8 presents the results of the association analysis. For each method the logarithm of the *p*-value of the different predictors is plotted along their position on the genome (this plot is also known as Manhattan plot).

Either methods highlight a region on chromosome 6 strongly associated with the phenotype. This region corresponds to the Major Histocompatibility Complex (MHC), and Human Leukocyte Antigen (HLA) class I molecules HLA B27 belonging to this region have been identified as a genetic risk factor associated with ankylosing spondylitis [38]. Our method SASA succeeds in detecting this risk locus with a good precision, 64 aggregated-SNPs variables are significantly associated with the phenotype compared to 602 significantly associated SNPs with the standard SMA approach.

**Fig. 9:**
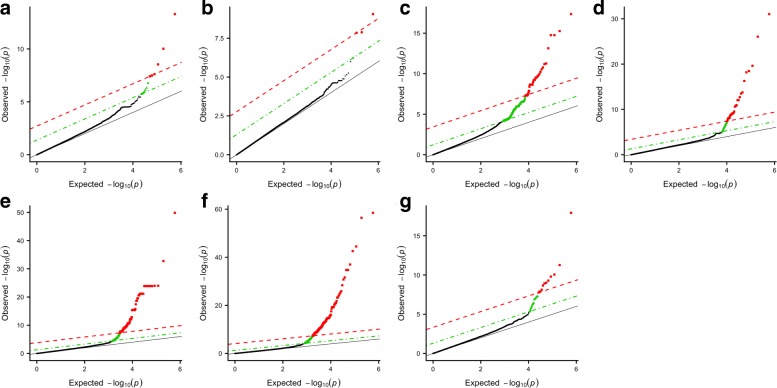
Q-Q plots of group-based genome-wide analysis on WTCCC data using the SASA approach. For each Manhattan plot, the Benjamini-Hochberg (BH) threshold is represented by the green dotted line and the Bonferroni threshold by the red dashed line. (**a**) Bipolar disorder - 13 significant clusters of SNPs; (**b**) Coronary arthery disease - 4 significant clusters of SNPs; (**c**) Inflammatory bowel disease - 356 significant clusters of SNPs; (**d**) Hypertension - 47 significant clusters of SNPs; (**e**) Rheumatoid arthritis - 202 significant clusters of SNPs; (**f**) Type I diabetes - 358 significant clusters of SNPs; (**g**) Type II diabetes - 28 significant clusters of SNPs

For the analysis of the WTCCC datasets, we represent the results, in Fig. 9, by plotting the expected *p*-value against the observed *p*-value (this a plot is known as Quantile-Quantile plot). We perform the analysis using our approach SASA only.

## Discussion

Overall,accounting for the linkage disequilibrium structure of the genome and aggregating highly-correlated SNPs is seen to be a powerful alternative to standard marker analysis in the context of GWAS. In terms of risk prediction, our algorithm proves to be very effective at classifying individuals given their genotype, while in terms of the identification of loci, it shows its ability to identify genomic regions associated with a disease with a higher precision than standard methods.

Is is also worth mentioning that our algorithm can also accomodate imputed variables as imputation in GWAS uses the Linkage Disequilibrium between variables to improve the coverage of variants. Our method being based on LD to define groups of common variants, we expect the group structure not to be impacted by imputation.

In this work we propose a four-step method explicitly designed to utilize the linkage disequilibrium in GWAS data. Our method combines, on the one hand, unsupervised learning methods that cluster correlated-SNPs, and on the other hand, supervised learning techniques that identify the optimal number of clusters and reduce the dimension of the predictor matrix. We evaluated the method on numerical simulations and real datasets and compared the results with standard single-marker analysis and group-based approaches (SKAT*tree* and SKAT*notree*). We remarked that the combination of our aggregating function with a ridge regression model leads to a major improvement in terms of predictive power when the linkage disequilibrium structure is strong enough, hence suggesting the existence of multivariate effects due to the combination of several SNPs. These results remained consistent across two applications involving several binary traits (WTCCC and ankylosing spondylitis datasets).

In terms of the identification of associated loci in different simulation scenarios, our method demonstrates its ability to retrieve true causal SNPs and/or clusters of SNPs with substantially higher precision coupled with a good power. On real GWAS data, our method has been able to recover a genomic region associated with ankylosing spondylitis (HLA region on chromosome 6) with a higher precision than standard single-marker analysis.

To improve our method further, while taking into account structured input variables in GWAS, there are different avenues that may be explored. One avenue would involve highlighting potential non-linear relationships between aggregated-SNPs and a response phenotype. This could be done by making use of the continuous nature of aggregated-SNPs variables (in contrast to the ordinal nature of single SNP variables), by using generalized additive models [39], and by performing non-linear regression using natural polynomial splines. In addition, whereas we evaluated our method for binary traits (case-control phenotype), a possible extension might include quantitative non-binary traits (i.e., using a ridge regression model instead of logistic ridge regression).

## Abbreviations

FAIS: Fast automatized interval search
GWAS: Genome-wide association studies
HLA: Human leukocyte antigens
LASSO: Least absolute shrinkage and selection operator
LD: Linkage disequilibrium
MAF: Marker allele frequency
MHC: Major histocompatibility complex
ROC: Receiver operating characteristic
SASA: Single aggregated-SNP analysis
SNP: Single sucleotide polymorphism
SMA: Single marker analysis
SKAT: Sequence kernel association test
WTCCC: Wellcome trust case-control consortium
